# Effectiveness of dimeticone oils versus sodium carbonate solution in the treatment of tungiasis in Kenya: a non-inferiority randomised trial

**DOI:** 10.1186/s41182-026-00909-7

**Published:** 2026-03-12

**Authors:** Kana Suzuki, Yasuhiko Kamiya, Chris Smith, Satoshi Kaneko, Juma Vitalis, Obino Tai, Abigael Osendi, Asiko Ongaya, Evans Amukoye

**Affiliations:** 1 School of Tropical Medicine and Global Health, Nagasaki University, Nagasaki, Japan; 2 Nursing Course, School of Medicine, Yokohama City University, Yokohama, Japan; 3 Department of Clinical Research, Faculty of Infectious and Tropical Diseases, London School of Hygiene and Tropical Medicine, London, UK; 4 Department of Ecoepidemiology, Institute of Tropical Medicine, Nagasaki University, Nagasaki, Japan; 5 Department of Health Services, Vihiga County, Vihiga, Kenya; 6 Kenya Medical Research Institute, Nairobi, Kenya

**Keywords:** Disease vectors, Treatment switching, Public health

## Abstract

**Background:**

Tungiasis is a cutaneous parasitic disease caused by the female flea *Tunga penetrans*. The World Health Organization recommends two-component dimeticone (NYDA^®^) as the sole treatment for tungiasis; however, this topical medication is not available in Kenya. In western Kenya, sodium carbonate has been adopted as a traditional village-based treatment. A pilot study found that the proportion of dead fleas on day 7 was higher with NYDA^®^ treatment than that with 5% sodium carbonate treatment (87% vs. 64%, respectively). This study was aimed at assessing the 11-day cure rates of tungiasis by comparing the efficacy of sodium carbonate and NYDA^®^ treatments in Vihiga County, Kenya.

**Methods:**

A randomised, observer-blinded, non-inferiority trial was conducted, with the non-inferiority margin set at 10%. A total of 160 eligible children with 941 flea infections were matched and randomised. The number of lesions per child per foot ranged from 1 to 10, with a median of 5 lesions. Each participant received both treatments, with one treatment applied to each foot. Health conditions, including inflammation scores and adverse events, were recorded. Observations were recorded on days 3, 5, 7, 9, and 11 using a digital microscope to determine flea viability.

**Results:**

Data from 157 children aged 4–15 years were analysed, comprising a total of 843 lesions. On day 11, the proportion of dead fleas was 88% for NYDA^®^ and 77% for 5% sodium carbonate solution (*p* < 0.05). No significant differences were observed in inflammation scores or symptoms such as pain and itchiness between the two treatments.

**Conclusions:**

This study demonstrated that 5% sodium carbonate did not meet the non-inferiority margin compared with NYDA^®^ in treating tungiasis. Nevertheless, in settings where NYDA^®^ is not accessible, it may still be considered an alternative.

*Trial registration* This study was registered with UMIN-CTR (Trial ID: UMIN000044320; reception desk number: R000050621) on 28 May 2021.

**Supplementary Information:**

The online version contains supplementary material available at 10.1186/s41182-026-00909-7.

## Background

Tungiasis is a cutaneous parasitic disease caused by the female sand flea *Tunga penetrans* [1]. Tungiasis is endemic in tropical and subtropical regions, with a high burden in sub-Saharan Africa, where it has been recognised as a Neglected Tropical Disease (NTD) by the World Health Organization (WHO). The WHO emphasises the need for further research to better estimate the global burden and distribution of this disease [2].

*Tunga penetrans* is a flea species whose unfertilised females penetrate the host’s skin and remain embedded until their death after 4–6 weeks [3, 4]. In homes with unsealed earthen floors, the sand flea life cycle can be completed indoors within sleeping areas [5, 6]. In Kenya, tungiasis has been linked to environmental, socioeconomic, and behavioural factors, including poor housing, low socioeconomic status, limited disease awareness, low health-seeking behaviour, and proximity to animals [7–9].

As a result, tungiasis remains a significant public health problem in impoverished communities, particularly in South America and sub-Saharan Africa [10]. Among skin-related neglected tropical diseases (skin NTDs), scabies has been relatively well documented in Kenya, with community-based studies reporting a prevalence of approximately 8.3% [11]. In contrast, epidemiological data on tungiasis remain limited, despite its substantial impact on vulnerable populations. Elson et al. [12] reported a varied prevalence for tungiasis in Kenya, ranging from 0.08% to 3.24%.

The disease shows a characteristic age-specific prevalence, peaking in children aged 5–14 years and in older adults [13]. Although rarely fatal, the disease can substantially impair quality of life. Infected children experience severe itching and pain, leading to increased absenteeism and poorer academic performance than their peers [14–16].

### Current treatment landscape

Traditional village-based treatment often involves surgical extraction of embedded fleas using improvised tools such as needles, safety pins, or pointed wood [17]. These methods are painful, time-consuming, and associated with risks of secondary infections, including tetanus, HIV, and hepatitis B and C, which are prevalent in sub-Saharan Africa [18, 19].

Kenyan National Policy Guidelines [20] recommend several agents for the treatment of tungiasis, such as Savlon, potassium permanganate, and hydrogen peroxide. In addition, topical agents commonly referred to as flea repellents, such as DEET, Zanzarin, neem and coconut oil preparations, jojoba oil, and aloe vera extract, are described as therapeutic options once infestation has occurred. Low-viscosity silicone oils (dimeticone) are also strongly endorsed given their efficacy and safety [21]. However, although various clinical trials have been conducted, sustainable treatment options remain limited. Ivermectin and metrifonate have proven ineffective [22], and dimethicone remains largely unavailable in endemic communities [21, 23].

### Alternative treatments

In Kenya, communities have traditionally used an herbal lotion made from neem seeds and coconut oil to treat tungiasis [15]. A study in Kilifi County found that although a 20% neem–coconut oil solution did not outperform potassium permanganate in flea mortality within 7 days, it led to better secondary outcomes, such as reduced acute pathology and higher odds of children being pain-free [15]. Another study in Muranga County tested 100% coconut oil [24], but owing to the short observation period, it remained unclear whether flea mortality was due to treatment effects or the fleas’ natural life cycle.

Unlike in East Kenya, where coconut oil is commonly used, communities in western Kenya, particularly Siaya County, rely on sodium carbonate—locally known as soda ash or Magadi. Sodium carbonate is a naturally occurring compound used in various industries, including food preparation and detergent production. Sodium carbonate is inexpensive, with 50 g costing approximately 10 Ksh (0.1 USD) in local markets. A 2016 study in Siaya County suggested its effectiveness against tungiasis [25], but the study lacked practical comparisons, had a limited observation period (only day 1), and did not assess safety.

### Rationale for this study

The two-component dimethicone formulation is the only tungiasis treatment recommended by the WHO and is included in Kenya’s national guidelines [10, 20]. A study in Kenya found that applying two-component dimethicone to the feet and ankles killed 78% of embedded fleas within 7 days [23]. However, its high cost—approximately $21 for 50 mL in European markets [26]—and limited availability make it impractical for widespread use in Kenya.

To explore alternative treatments, we conducted a pilot study [27] comparing 5% sodium carbonate solution and NYDA^®^ (dimethicone). The results showed that the cure rate on day 7 was significantly higher with NYDA^®^ (87%) than with sodium carbonate (64%), although both treatments exhibited similar safety profiles. Despite the superior efficacy of NYDA^®^, sodium carbonate remains a promising alternative owing to its affordability and local availability.

Building on these findings, the present study extends the observation period to 11 days to further assess the efficacy and safety of both treatments, with a particular focus on the feasibility of sodium carbonate as a cost-effective, locally available alternative to dimethicone.

### Hypothesis

The cure rate of 5% sodium carbonate would be no more than 10% lower than that of NYDA^®^ for the treatment of tungiasis.

### Objectives

#### Main objective

To assess the efficacy and safety of 5% sodium carbonate and dimethicone in the treatment of tungiasis among children in Kenya.

#### Specific objectives

1. To compare the cure rates of the two treatments within 11 days: 5% sodium carbonate and NYDA^®^.
2. To assess the safety of the two treatments by determining the incidence of adverse effects.
3. To assess the impact of both treatments on inflammation scores.
4. To assess the acceptability of the two treatments within the community.

## Methods

The study design and procedures were described previously [28]. Briefly, the present trial was conducted in a different study area and with a modified participant age range compared with those in the original protocol.

### Study design

This study was a randomised, observer-blinded, non-placebo-controlled, non-inferiority trial with two treatment arms: 5% sodium carbonate and NYDA^®^ for the treatment of tungiasis. All participants received both treatments, with one applied to each foot. Therefore, allocation to treatment groups was not possible with participant and intervener masking. The study period was from January to June 2024.

### Study area

The study was conducted in Vihiga County, which consists of five sub-counties: Luanda, Emuhaya, Hamisi, Sabatia, and Vihiga. Based on the Ministry of Health surveillance reports, Hamisi and Emuhaya sub-counties were first purposively selected because they reported the highest number of tungiasis cases. A previous study reported a 21.5% prevalence in parts of Vihiga County [29]. The study took place at 15 public primary/ECD schools in the most affected regions in these two sub-counties.

### Study population

The study recruited children aged 4–15 years living in Hamisi and Emuhaya sub-counties in Vihiga County, Kenya. Children were included if diagnosed as affected with *Tunga penetrans* and presenting with symptoms and signs of tungiasis, identified as Fortaleza classification stages II or III [3].

### Eligibility criteria

1. Children aged 4–15 years who were infected with tungiasis in Vihiga County, Kenya.
2. Children and caregivers provided written assent/fingerprints and consent to participate in this study.
3. Children with more than one viable flea in both feet and 1–5 embedded fleas in both feet were enrolled.

### Exclusion criteria

1. Children infected with > 20 fleas, who required immediate evidence-based treatment (treated promptly with NYDA^®^).
2. Children or caregivers who declined to participate.
3. Children with another pruritic skin condition on their feet.

### Outcome measures

The primary endpoint was the cure rate at day 11 after being treated with either 5% sodium carbonate solution or NYDA^®^. The embedded fleas were assessed at baseline and on days 3, 5, 7, 9 and 11 using a digital handheld microscope with 5-megapixel optical resolution. Four viability signs were evaluated: expulsion of eggs, excretion of faecal thread, excretion of faecal liquid, and pulsations/contractions. An embedded flea was considered to be non-viable when none of the four viability signs were detected during 10 min of observation. The study images are provided in Additional file 1.

Secondary endpoints were adverse effects (nausea/vomiting, arthralgia, fever, headache, fatigue/malaise, myalgia, and chills), acute pathology scores (pain, itching, erythema, warmth, oedema, desquamation, fissure, suppuration, ulcer, abscess, and lesions in clusters), and differences in feeling and easiness of children during the 11 days after the treatments.

### Assessors and clinical oversight

Lesion observation and assessment of flea viability (live or dead) were conducted by four laboratory technicians who completed a 2-day standardised training program, including hands-on practical exercises. Clinical monitoring of participants was carried out by four nurses or clinical officers who received the same training. Two team members with prior experience in the pilot study [27] provided overall supervision and follow-up. Independent monitoring was performed by an external expert with clinical and monitoring experience in tungiasis. In addition, a study physician was assigned to the trial and consulted as needed regarding participants’ clinical conditions.

### Sample size

#### Sample size calculation

Based on the hypothesis that 5% sodium carbonate has a 70% cure rate [25] and NYDA^®^ an approximate 78% cure rate [23], we calculated the sample size assuming both cure rates to be 80%. The sample size was calculated using the Sample-Size Formula [30], assuming the non-inferiority margin is chosen to be 10% [28]. We also assumed that the true mean cure rates of the treatment agent (sodium carbonate) and the active control (NYDA^®^) were 80% (pT = 0.80) and 80% (pC = 0.80), respectively. A 90% power (1–*β* = 0.9) at the 5% level of significance (*α* = 0.05) with equal allocation (*k* = 1) was achieved to demonstrate non-inferiority for the clinical cure assessment of efficacy. Given that one to five lesions per foot in one individual child were studied, intra-class correlation coefficient (ICC) (1, k) and *k* = 2–5 for the intra-rater reliability was estimated at 0.5. As the cluster size was 2, the design effect (DE) was calculated as 1 + (2 − 1) × 0.5 = 1.5. A total of 920 (460 per arm) lesions comprised the sample size, considering a 10% loss at follow-up.

### Sampling and recruitment

A multi-stage sampling method was used in this study.

Stage 1 involved sampling of sub-counties, stage 2 involved sampling of schools, stage 3 involved selecting participants, and stage 4 involved sampling embedded sand fleas.

#### Stage 1: sampling of sub-counties

Hamisi and Emuhaya sub-counties were selected because of the highest number of reported cases of tungiasis in Vihiga County.

#### Stage 2: sampling of schools

Primary schools and ECD centres in the study area were ranked according to the number of cases reported to the Health Department. We visited the schools in the most reported regions with the highest number of affected students in order.

#### Stage 3: sampling of participants

Within each school, children aged 4–15 years were selected in descending order of grade. At the beginning of the study, a sealed envelope was used for randomising the two treatments. The sealed envelopes had either ‘Right foot: NYDA^®^, Left foot: 5% sodium carbonate’ or ‘Right foot: 5% sodium carbonate, Left foot: NYDA^®^’ written in them, with a participant number. Before randomisation, lesions were sampled to avoid selection bias.

Children were informed not to manipulate the lesions during the next 11 days. Children whose skin problems were suspected to be tungiasis by teachers, caregivers, and healthcare workers, including Community Health Volunteers (CHVs), were screened for eligibility to participate in this study. Eligible children and their caregivers were then contacted by the local co-investigator and field assistants to ask if they were willing to participate in the study after explaining the study objectives and procedures. Informed consent from the caregivers and assent from the children were obtained after both agreed to participate. Participants were randomly selected until the point of saturation was reached.

#### Stage 4: sampling of embedded sand fleas

For each participant, one to five embedded sand fleas per foot were selected by the trained assessor to ensure an even distribution of lesions. Because it is difficult to distinguish the characteristics of individual lesions [21], we limited the number of lesions. According to the Fortaleza classification [3], the growth stage of the target lesions was Stage II or III. Only lesions that could be clearly distinguished from one another were included in the study.

### Randomisation, allocation, and blinding

All study participants received 5% sodium carbonate and NYDA^®^, one treatment per foot. Randomisation was not performed at the participant level. Instead, a within-participant design was used, and simple randomisation of treatment allocation to the left or right foot was performed by the participants themselves using sealed envelopes. The interventions were administered by trained nurses or clinical officers. Blinding the research assistant administering the treatment was not possible because 5% sodium carbonate required soaking the foot, whereas NYDA^®^ was applied to each lesion. Only the observers were blinded. Outcome assessment was conducted by independent observers who were blinded to treatment allocation.

### Recruitment strategy

Children eligible for the study were recruited from their school. A trained research assistant briefly explained the research, and once consent was obtained, the research assistant visited the home with the child. The informed consent documents were written in the local language.

### Intervention

According to the pilot study [27], a longer observation period was required to better understand the outcome; therefore, in this study, the primary and secondary observation periods were set at 11 days.

All procedures were conducted at school in an empty classroom to ensure privacy and avoid external interference. After washing the affected area with soap and clean water, both treatments were applied, one to each foot. One foot was soaked in the 5% sodium carbonate solution, which has > 99% purity. The solution was made by dissolving 250 g of sodium carbonate in 5 L of warm potable water within a deep basin. The affected parts were soaked in the solution for 15 min. The other foot was treated with NYDA^®^, applied directly to the affected area. The reagent was drawn into a 5-mL syringe to which a flexible tube was affixed. Three drops were applied to the targeted area. One drop corresponds to approximately 0.05 mL of NYDA^®^, and the procedure was repeated three times within 10 min to ensure that a maximum amount of NYDA^®^ entered the abdominal cone of the parasite. Lesions for outcome assessment were predefined before intervention, and only these lesions were followed throughout the study period. Outcomes were evaluated at predefined follow-up time points on days 3, 5, 7, 9, and 11 after treatment initiation. If the child was wearing sandals, we provided a pair of socks, and if they were barefoot, we provided a pair of sandals and socks to prevent reduction of treatment effectiveness; thereafter, all participants received identical treatment, and baseline footwear was not assessed. On the final day of the study, all children received treatment with NYDA.

### Data

Data were recorded in REDCap. For mapping, the selected lesions were drawn on standardised paper forms, and their pictures were also taken in the field. In addition, background information on participants was collected using a structured questionnaire.

As this was a school-based study, consent was first obtained from each school. Informed consent was then collected from each participant. For lesions not studied, NYDA^®^ was applied later if desired. During treatment, skin symptoms were monitored until the final decision on the 11th day using a checklist. Lesions were counted (alive, dead), and signs of flea viability were identified using a handheld digital microscope. Participants were observed and queried for adverse effects, and the severity score was used to measure pathology for acute tungiasis disease, which included a pain and itching score [31]. The feeling and easiness of each treatment were assessed by questionnaire. Trained nurses and clinical officers monitored the participants and interviews under a doctor’s guidance at treatment initiation and at the final decision.

### Data management

No participant data remained on hard drives once data entry was confirmed. Encrypted data from smartphones were automatically uploaded to a secure and backed-up server. Data collected using REDCap were sent via the web to the REDCap Server for storage. All data were stored in encrypted files on a server at KEMRI protected by a password. Any hardcopy data, including the signed consent and assent forms, were stored securely in a locked cabinet in the Nagasaki University Mbita Research Office. They will be shredded after 5 years.

### Statistical analysis

As the primary outcome of this study was binary categorical data that recruited multiple lesions per person, a generalised linear mixed-effects model was used to analyse the paired data, with the individuals treated as random factors. The per-protocol set was adopted in this study to compare local practices with the standard WHO-recommended treatment. Only treatment-adhered study participants were analysed, and instances of flea removal were excluded such that each participant had no missing values for flea infections. The log-rank test was performed to compare non-viability trends between the two treatments. The Wilcoxon signed-rank test was performed to compare the differences in terms of safety of both treatments. All data were analysed using EZR version 1.54, which is a graphical user interface for R version 4.0.3. More precisely, it is a modified version of R Commander designed to add statistical functions frequently used in biostatistics [32].

### Adverse events/effects

The study participants were carefully examined on days 1, 3, 5, 7, 9, and 11, with nurses or clinical officers specifically enquiring about symptoms such as blisters, soreness, fever, and headache. During each examination, the participants’ temperature, blood pressure, and pulse rate were recorded. Adverse events were recorded in the adverse events log.

### Ethics statement

This study was approved by the Medical Research Ethics Review Board of Nagasaki University, Japan (Reference Number: NU_TMGH_2022_177_1). This study was approved by the Scientific and Ethics Review Committee of Kenya Medical Research Institute (reference number: KEMRI/SERU/CRDR/070/4420). This study conformed to the principles of the Declaration of Helsinki. Approval was also obtained from The National Commission for Science, Technology and Innovation (Reference Number: NACOSTI/P/22/19550) of Kenya. This study was registered with UMIN-CTR (UMIN trial ID: UMIN 000044320; reception desk number: R000050621) on May 28, 2021, which is recognised internationally as follows: https://center9.umin.ac.jp/FAQ/en/UMIN-CTR_e/#faq-5_1.

Patient and public involvement was incorporated during data collection and in the interpretation of results. Their engagement helped to ensure that the findings were grounded in participants’ experiences and relevant to the local context.

## Results

### Participants

**Fig. 1:**
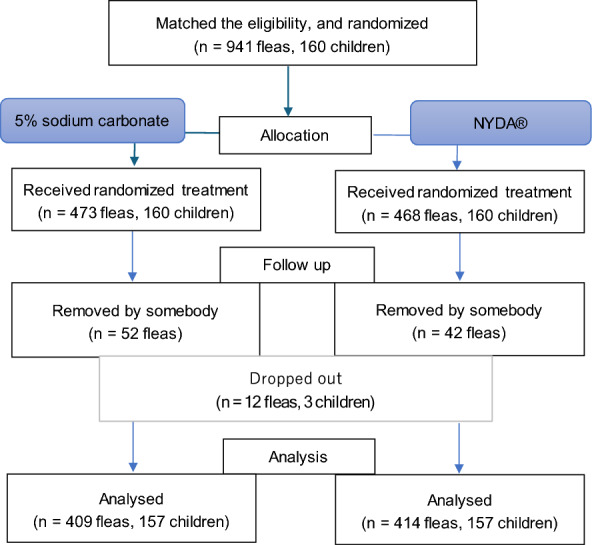
Flow diagram of the trial

In total, 941 flea infections in 160 participants were described during the initial screening, but only 823 flea infections were included in the final analysis after excluding 94 owing to flea removal and three children who dropped out (12 flea infections) (Fig. 1). Treatment-adherent refers to participants who completed the scheduled treatment and follow-up assessments. If a participant had multiple lesions and one was removed manually, the remaining lesions were still included in the analysis. All 157 children who underwent randomisation completed the study, with a mean age of 9.95 years. The majority were male (69.0%). Individual and socioeconomic risk factors are listed in Additional file 2.

Most children wore moderately maintained clothes (72.0%), while 15.9% wore torn clothes. Footwear observation showed that 14.6% of the children were barefoot, and 66.9% wore worn-out shoes. For school footwear, 14.0% of children had no shoes, while only 34.4% wore closed shoes. A complete school uniform was observed in 29.2%, while 6.4% had none.

Regarding housing conditions, 97.5% lived in homes with iron sheet roofs, and 82.2% had mud walls. Farming (54.6%) and housework (25.2%) were the main parental occupations. Most children (61.1%) slept on the floor. Soap was used by 89.8%, and 48.4% reported daily washing.

Ownership of domestic animals was common: 82.2% of participants reported owning chickens, 71.2% owned cows, 30.1% had dogs, 27.6% had cats, 14.1% had goats, and 2.5% each owned pigs and sheep.

When asked about their knowledge of tungiasis, 96% answered correctly about the causes, and 100% answered correctly about the symptoms.

### Cure rates (percentage of dead fleas)

**Fig. 2:**
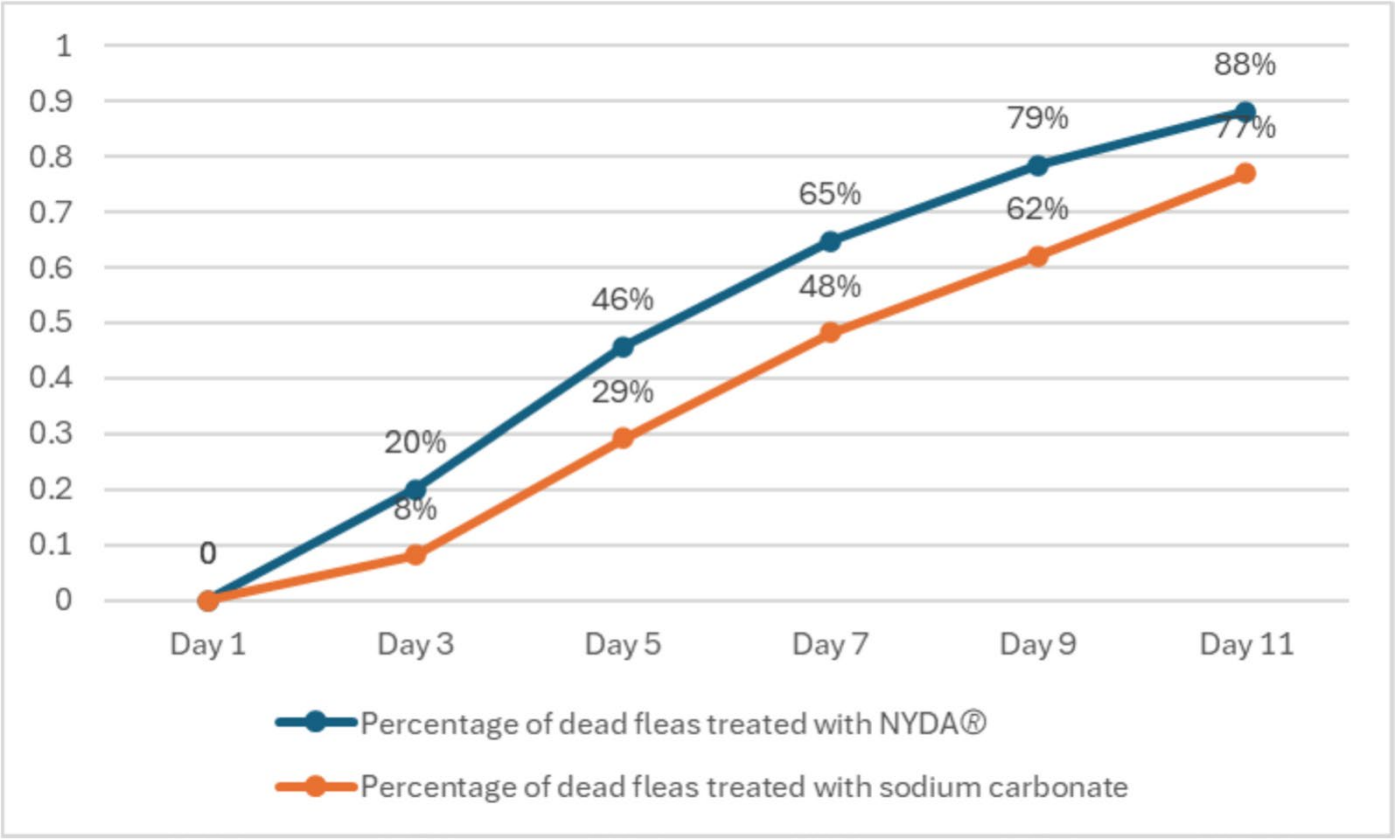
Trend in the percentage of dead fleas over the observation period

On day 11, the proportion of dead fleas was 88% in the NYDA^®^-treated group and 77% in the 5% sodium carbonate-treated group (*p* < 0.01, Fig. 2). A Chi-square test demonstrated a significant difference between these two treatments (*p* < 0.05).

### Safety

**Fig. 3:**
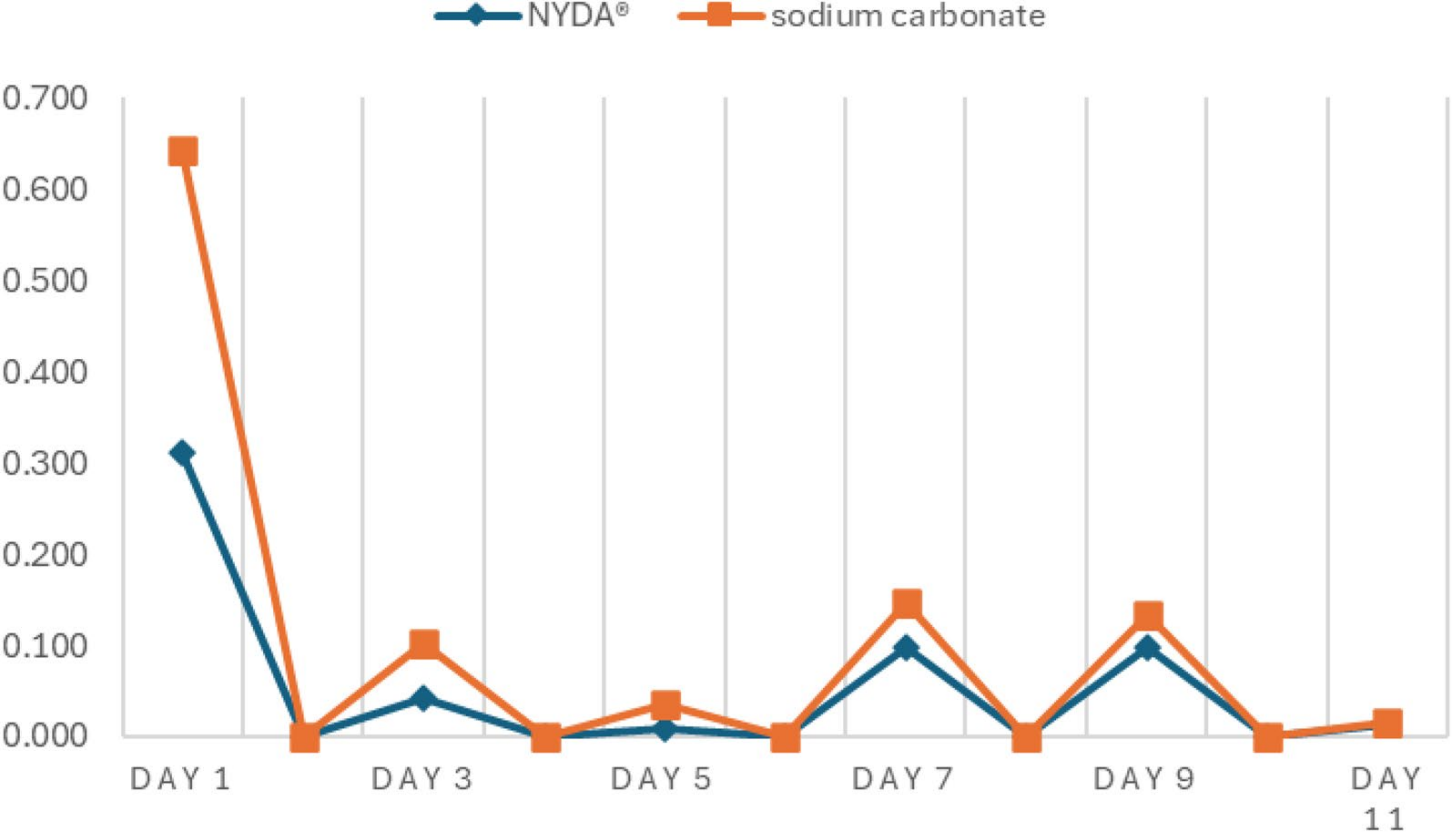
Inflammation score

Changes in the average inflammation scores over time are shown in Fig. 3. Both treatments showed a transient increase in inflammation scores on day 7, followed by a declining trend towards day 11. NYDA^®^ treatment consistently resulted in lower scores than sodium carbonate treatment on all observation days. Inflammation peaked on day 7 for both treatments. Table 1 shows the proportion of participants who reported itching and pain during the 11-day period. Complaints of both symptoms peaked on day 5 for both treatment groups, but the difference was not statistically significant. However, one case of pain was reported in a foot treated with sodium carbonate. Upon examination by a medical doctor (MD), redness was observed, and the area was cleansed with clean water. The condition was attributed to a pre-existing cut on the child’s foot, and the case was excluded from the study. Monitoring continued, and on day 7, an MD conducted a follow-up evaluation. The child’s condition was found to be good, the redness had resolved, and the lesion was confirmed to have healed.

**Table 1:**
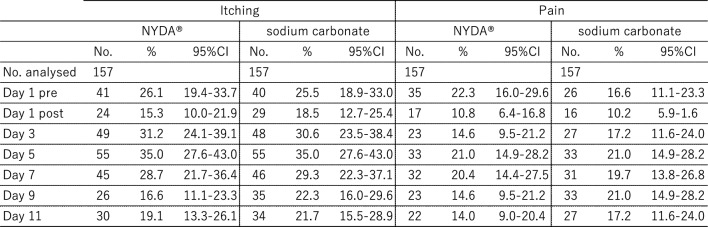
Proportion of itching and pain reports

### Feelings, ease, and participant choice of favourite treatment

**Fig. 4:**
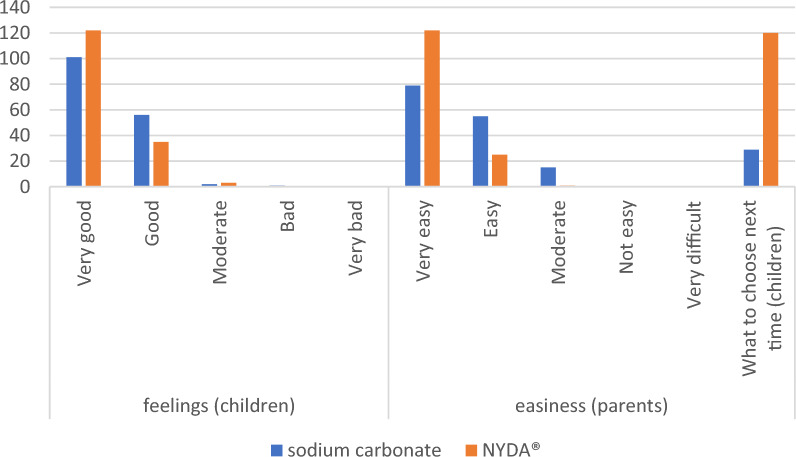
Feelings, easiness, and participants’ choice of favourite treatment

Children were asked to describe their feelings during the treatments, and parents were asked about the ease of applying these treatments (Fig. 4). None of the children reported negative feelings, and no parents rated the treatments as difficult to apply. A total of 157 children reported very good or good feelings toward both treatments. Regarding feasibility, 134 parents (48%) rated sodium carbonate as very easy or easy to use, while 147 parents (52%) gave the same rating for NYDA. When asked about future preference, 120 children (81%) chose NYDA, whereas 29 children (19%) preferred sodium carbonate.

## Discussion

This study introduced three key modifications compared with the pilot study: (1) the study participants, (2) observation period, and (3) the study location.

### Study participants

In the present study, a higher proportion of participants were boys. This finding is consistent with a recent systematic review [33], which clearly identified male sex as a risk factor for tungiasis (adjusted odds ratio 1.53, 95% CI 1.05–2.01). The review suggests that this difference may be explained by the observation that boys more frequently play in unpaved streets, resulting in higher exposure rates, whereas women tend to have a lower prevalence due to less frequent outdoor activity. Importantly, this disparity was not attributed to differences in health-oriented behaviours after infection. Although observational data on behaviour were not collected in the present study, tungiasis was more commonly observed among barefoot boys in the participating schools. It is also plausible that frequent barefoot movement in areas surrounding livestock contributes to increased exposure risk among boys.

Regarding follow-up feasibility, previous school-based studies have reported varying degrees of loss to follow-up. Thielecke et al. [21], who also studied school children, reported that approximately half of the participants were not accessible at follow-up. Differences in school attendance rates across regions may also have contributed to these variations in follow-up rates. In contrast, Elson et al. [15] reported a loss to follow-up of 3 out of 47 children. In this context, the loss of only three participants out of 160 in the present study indicates that the recruitment strategy and study setting were highly feasible and suitable for conducting intervention research in school settings.

### Observation period

Extending the observation period from 7 to 11 days, as suggested by the pilot study, reduced the difference in cure rates between the two treatments. However, NYDA^®^ remained significantly superior. As expected, the therapeutic effect of sodium carbonate emerged more slowly than that of NYDA®, further confirming its delayed efficacy. Extending the observation period beyond 11 days was deemed impractical, as it would make it difficult to distinguish treatment effects from natural flea mortality. This decision aligns with the Fortaleza classification [3], which states that stage II and III lesions—the eligibility criteria for this study—naturally resolve within approximately two weeks.

### Study location

The study site was moved from Homa Bay County to Vihiga County, where a higher case prevalence made it a more suitable research setting. Additionally, conducting the study in two different regions provided a broader perspective on cure rates. When comparing the day 7 cure rates with previous studies, our findings indicate lower efficacy for both treatments. Prior studies reported NYDA® cure rates of 78%, 87%, and 97% [21, 23, 27] and sodium carbonate at 64% [27]. Given that the study methodology remained largely unchanged, regional differences could explain the variations in outcomes. As shown in Additional file 3, previous studies were conducted in Uganda and Kenya, specifically, eastern Uganda and central/western Kenya. Although these areas are geographically close within Africa, genetic variations in sand flea populations may contribute to differing treatment outcomes.

Furthermore, when comparing the day 7 cure rates of other single-application tungiasis treatments, the results of this study suggest a relatively higher efficacy. In this study, the cure rate was 65% for NYDA^®^ and 48% for 5% sodium carbonate. In contrast, previously reported cure rates include a combination of coconut oil and neem oil at 30% [15] and KMnO₄ at 39% and 40% [15, 21]. The 11–17% difference in cure rates between NYDA^®^ and sodium carbonate suggests that sodium carbonate on days 7–11 could still be a viable option in resource-limited settings.

With a sufficient sample size and an extended observation period, this study confirms the limitations of sodium carbonate while consistently demonstrating the superior efficacy of NYDA^®^ across two different locations, despite the absence of genetic data on sand fleas. Future research should focus on optimising sodium carbonate formulations, investigating its mechanism of action, or identifying alternative cost-effective treatments suitable for endemic regions.

This study demonstrated that 5% sodium carbonate did not meet the non-inferiority margin compared with NYDA^®^ for treating tungiasis. However, it has a certain potential as an alternative treatment given that it yields the second-highest cure rates compared with other treatments, as demonstrated by clinical trials. In the present study, a higher-purity sodium carbonate than that used in a prior pilot study [27] was applied, which may partly explain the observed improvement in cure rates. Conversely, because one participant reported pain during treatment, sodium carbonate should be avoided in patients with other skin problems. With appropriate patient selection and precautions, sodium carbonate could be considered as an additional treatment option for tungiasis in resource-limited settings.

The main limitation of this study lies in the reliability of the safety data, such as pain, itching, and inflammatory scores. These outcomes were not assessed at the individual lesion level but were evaluated per foot, which may have reduced the sensitivity in the detection of lesion-specific inflammatory responses. In addition, because objective measures were not collected, whether the reported symptoms were directly ascribed to the treated target lesions remains unclear. Future studies should consider the use of devices such as thermography to obtain more objective data. A major strength of this study is that the intervention was compared against NYDA^®^, a standard treatment that has demonstrated cure rates exceeding 80%. In contrast, most previous superiority trials have employed KMnO₄, which typically achieves cure rates of around 40% [15, 34, 35]. NYDA^®^ has rarely been used as a control owing to its limited availability or the preference for demonstrating superiority. To our knowledge, this is the first non-inferiority trial conducted against the established standard treatment.

## Conclusions

An accessible, safe, and effective treatment for tungiasis has yet to be firmly established. In the meantime, healthcare providers and affected communities continue to rely on methods they consider appropriate, in the absence of standardised guidance [9]. It is therefore imperative that an affordable and widely accessible therapeutic option be approved globally as an alternative treatment without further delay. Future research should explore practical treatment approaches grounded in the realities of endemic settings and informed by direct field experience.

## Supplementary Information

Supplementary Material 1. Supplemental figure 1 Study images.

Supplementary Material 2. Supplemental table 1 Characteristics of study participants: Individual and Socioeconomic Risk Factors.

Supplementary Material 3. Supplemental figure 2 Map of the previous studies with NYDA and sodium carbonate.

## Data Availability

The datasets generated and/or analysed during the current study are not publicly available due to privacy and ethical restrictions, but de-identified data may be available from the corresponding author on reasonable request.

## Abbreviations

CHV: Community Health Volunteer
DE: Design effect
ICC: Intra-class correlation coefficient
MD: Medical doctor
NTD: Neglected tropical disease
WHO: World Health Organization
