# Supplementary material for: Effectiveness of dimeticone oils versus sodium carbonate solution in the treatment of tungiasis in Kenya: a non-inferiority randomised trial
Source: Trop Med Health. 2026 Mar 12;54:46. doi: 10.1186/s41182-026-00909-7 (PMC12980886; doi:10.1186/s41182-026-00909-7)
Supplement: Supplementary file 1 — Supplementary Material 1. Supplemental figure 1 Study images. [file 41182_2026_909_MOESM1_ESM.docx]

**
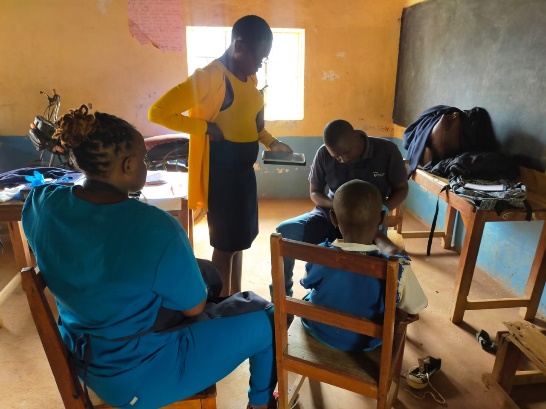

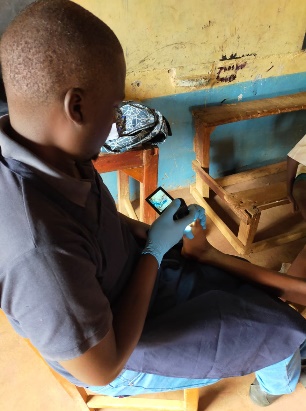
**

Supplementary material 1. Study images (left: the research team surrounding the participant; right: using a handheld microscope to assess flea viability)
