# Supplementary material for: Effectiveness of dimeticone oils versus sodium carbonate solution in the treatment of tungiasis in Kenya: a non-inferiority randomised trial
Source: Trop Med Health. 2026 Mar 12;54:46. doi: 10.1186/s41182-026-00909-7 (PMC12980886; doi:10.1186/s41182-026-00909-7)
Supplement: Supplementary file 2 — Supplementary Material 2. Supplemental table 1 Characteristics of study participants: Individual and Socioeconomic Risk Factors. [file 41182_2026_909_MOESM2_ESM.docx]

Supplementary material 1. Characteristics of study participants: Individual and Socioeconomic Risk Factors

|  | CATEGORIES | RESPONSES | NO. (%) |
| --- | --- | --- | --- |
|  | Age |  | mean 9.9 |
|  | Sex | Male | 108 (69.0) |
|  |  | Female | 49 (31.0) |
| Individual risk factors | Condition of uniform/other clothes | torn/rags | 25 (15.9) |
|  | (observation) | moderate | 113 (72.0) |
|  |  | smart | 18 (11.5) |
|  | Shoes wearing | none | 23 (14.6) |
|  | (observation) | open shoe | 80 (51.0) |
|  |  | closed shoe | 53 (33.8) |
|  | Condition of the footwear | worn out | 105 (66.9) |
|  |  | intact | 50 (31.8) |
|  | Shoes wearing for school | none | 22 (14.0) |
|  |  | open shoe | 79 (50.3) |
|  |  | closed shoe | 54 (34.4) |
|  | Presence of school uniform | None | 10 (6.4) |
|  |  | Partially | 100 (63.7) |
|  |  | Complete | 46 (29.2) |
| Socioeconomic risk factors | Material on the roof of the house | Grass or Makuti | 4 (2.5) |
|  |  | Iron Sheets | 159 (97.5) |
|  |  | Others | 0 (0) |
|  | Material of the walls in the house | stone | 0 (0) |
|  |  | mud | 134 (82.2) |
|  |  | grass or makuti | 1 (0.6) |
|  |  | cemented | 3 (1.8) |
|  |  | dung plastered | 23 (14.1) |
|  | Income sources of the parents | Housewife | 41 (25.2) |
|  |  | Farmer | 89 (54.6) |
|  |  | Fishing industry | 0 (0) |
|  |  | Small scale business | 16 (9.8) |
|  |  | Formally employed | 4 (2.5) |
|  |  | Others | 11 (6.7) |
|  | Sleeping place | bed | 57 (36.3) |
|  |  | floor | 96 (61.1) |
|  |  | others | 3 (1.9) |
|  | Using soap while washing | yes | 141 (89.8) |
|  |  | no | 15 (9.6) |
|  | Frequency of washing | Once in two days | 57 (36.3) |
|  |  | Once a day | 76 (48.4) |
|  |  | Twice a day | 6 (3.8) |
|  |  | Others: specify | 17 (10.8) |
| Domestic animals | Owning chicken | owning | 134 (82.2) |
|  |  | don't have | 29 (17.8) |
|  | Owning dogs | owning | 49 (30.1) |
|  |  | don't have | 114 (69.9) |
|  | Owning pig | owning | 4 (2.5) |
|  |  | don't have | 159 (97.5) |
|  | Owning cats | owning | 45 (27.6) |
|  |  | don't have | 118 (72.4) |
|  | Owning goats | owning | 23 (14.1) |
|  |  | don't have | 140 (85.9) |
|  | Owning sheep | owning | 4 (2.5) |
|  |  | don't have | 159 (97.5) |
|  | Owning cow | owning | 116 (71.2) |
|  |  | don't have | 47 (28.8) |
