# Supplementary figures and images for: Effectiveness of dimeticone oils versus sodium carbonate solution in the treatment of tungiasis in Kenya: a non-inferiority randomised trial
Source: Trop Med Health. 2026 Mar 12;54:46. doi: 10.1186/s41182-026-00909-7 (PMC12980886; doi:10.1186/s41182-026-00909-7)

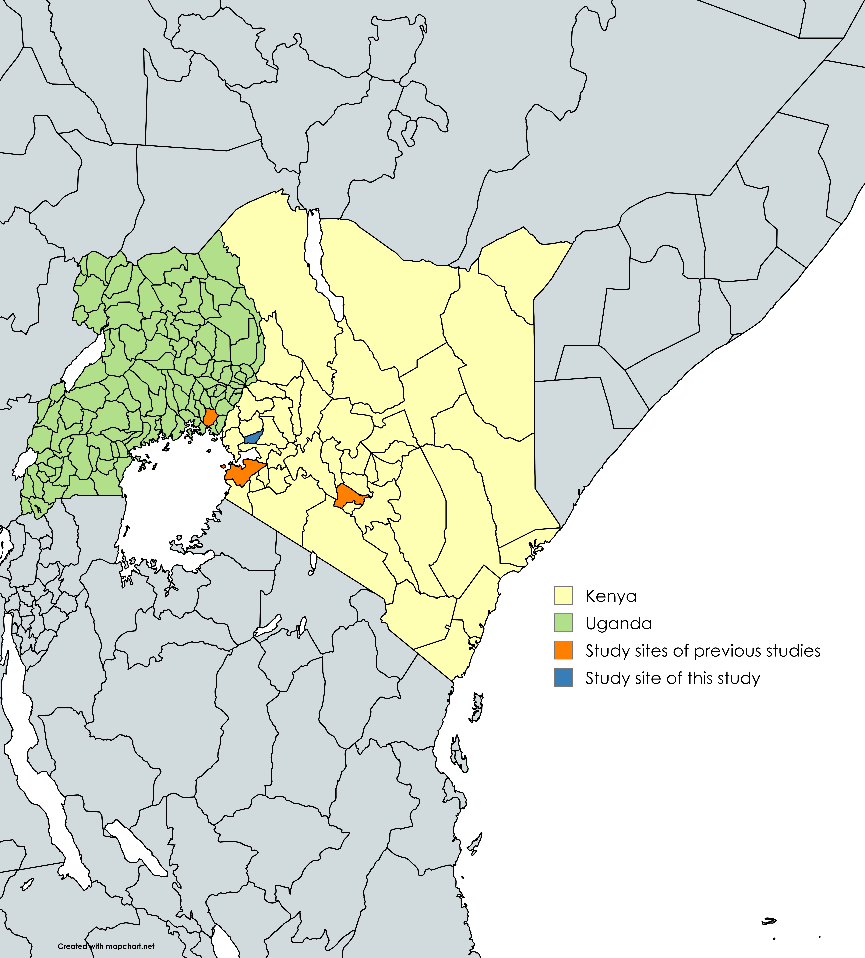


Supplementary material 3. Map of the previous studies with NYDA and sodium carbonate

Supplement: Supplementary file 3 — Supplementary Material 3. Supplemental figure 2 Map of the previous studies with NYDA and sodium carbonate. [file 41182_2026_909_MOESM3_ESM.docx]
